# Discriminating Active from Latent Tuberculosis in Patients Presenting to Community Clinics

**DOI:** 10.1371/journal.pone.0038080

**Published:** 2012-05-30

**Authors:** Gurjinder Sandhu, Francesca Battaglia, Barry K. Ely, Dimitrios Athanasakis, Rosario Montoya, Teresa Valencia, Robert H. Gilman, Carlton A. Evans, Jon S. Friedland, Delmiro Fernandez-Reyes, Daniel D. Agranoff

**Affiliations:** 1 Department of Infectious Diseases and Immunity and Wellcome Trust Centre for Clinical Tropical Medicine, Imperial College London, London, United Kingdom; 2 Division of Parasitology, Medical Research Council National Institute for Medical Research, London, United Kingdom; 3 Department of International Health, Johns Hopkins Bloomberg School of Public Health, Baltimore, Maryland, United States of America; 4 Associacion Benefica PRISMA, San Miguel Laboratorio de Investigacion de Enfermedades Infecciosas, Universidad Peruana Cayetano Heredia, Lima, Peru; 5 Faculty of Science and Philosophy, Universidad Peruana Cayetano Heredia, Lima, Peru; National Institute for Infectious Diseases (L. Spallanzani), Italy

## Abstract

**Background:**

Because of the high global prevalence of latent TB infection (LTBI), a key challenge in endemic settings is distinguishing patients with active TB from patients with overlapping clinical symptoms without active TB but with co-existing LTBI. Current methods are insufficiently accurate. Plasma proteomic fingerprinting can resolve this difficulty by providing a molecular snapshot defining disease state that can be used to develop point-of-care diagnostics.

**Methods:**

Plasma and clinical data were obtained prospectively from patients attending community TB clinics in Peru and from household contacts. Plasma was subjected to high-throughput proteomic profiling by mass spectrometry. Statistical pattern recognition methods were used to define mass spectral patterns that distinguished patients with active TB from symptomatic controls with or without LTBI.

**Results:**

156 patients with active TB and 110 symptomatic controls (patients with respiratory symptoms without active TB) were investigated. Active TB patients were distinguishable from undifferentiated symptomatic controls with accuracy of 87% (sensitivity 84%, specificity 90%), from symptomatic controls with LTBI (accuracy of 87%, sensitivity 89%, specificity 82%) and from symptomatic controls without LTBI (accuracy 90%, sensitivity 90%, specificity 92%).

**Conclusions:**

We show that active TB can be distinguished accurately from LTBI in symptomatic clinic attenders using a plasma proteomic fingerprint. Translation of biomarkers derived from this study into a robust and affordable point-of-care format will have significant implications for recognition and control of active TB in high prevalence settings.

## Introduction

Tuberculosis is the leading bacterial cause of death worldwide, with an estimated 8.8 million new cases of active disease and 1.6 million deaths per year [1]. Much of the burden of disease lies in the developing world, where annual incidence can reach 700 per 100,000 in certain regions [1]. New and unrecognised cases drive the epidemic, with transmission usually occurring before the index case is diagnosed. Multi-drug resistant cases and HIV co-infection further complicate control efforts [2]. Pulmonary TB is the most frequent clinical and transmissible manifestation of active disease. Rapid diagnosis and treatment are critical in the prevention of transmission.

The global burden of active TB occurs on a background of quiescent or latent TB infection (LTBI), affecting one third of the world’s population and a higher proportion of the population of TB-endemic area*s* [3]. Respiratory and constitutional symptoms overlapping with those of pulmonary TB are very common in communities where TB is endemic [4]. In this scenario the challenge is to distinguish symptomatic patients with active TB from those with latent disease but whose presenting symptomatology is attributable to some other infectious or inflammatory process. In terms of rapid diagnosis, sputum microscopy will only identify approximately 50% of patients with active pulmonary TB. Conversely, while the interferon gamma release assays (IGRAs) represent a major advance in the detection of latent TB, they cannot distinguish active TB from symptomatic patients with latent infection in this context [5], [6]. This overlap between LTBI, active TB and non-specific clinical manifestations presents a formidable obstacle to the rapid recognition of active TB and the timely and appropriate targeting of anti-TB chemotherapy or chemoprophylaxis. In practice this difficulty may give rise to 2 types of therapeutic error. In the first instance, erroneous diagnosis of active TB in a symptomatic patient with LTBI may result in inappropriate administration of full course TB treatment. Conversely, offering chemoprophylaxis to a patient with supposed LTBI in whom active TB has not been recognized, will drive emergence of drug resistance.

Pulmonary TB is characterised by granuloma formation, caseation and ultimately cavitation, reflecting a complex interplay between distinctive components of the innate and acquired immune response and the pathogen [5]. Traditional serological analysis of single circulating proteins is notoriously unreliable for TB diagnosis [7]. In contrast, patterns of circulating proteins could provide an accessible readout of pathophysiological status. Discovery of such discriminatory biomarkers could open the way for the development of new point-of-care tests based on a lateral flow format such as dipsticks.

Proteomic analysis using Surface Enhanced Laser Desorption Ionisation Time of Flight (SELDI-ToF) mass spectrometry is a high throughput profiling methodology, which enables rapid comparison of protein patterns from large numbers of patients. The conceptual approach employed in the present study is termed proteomic fingerprinting. It is based on the principle that distinctive combinations of circulating proteins characterize different disease states. This strategy has been applied to the discovery of discriminatory proteomic patterns for a range of diseases including cancer [8], vascular disease [9]–[11] and infectious diseases [12]–[15]. Previously, we have demonstrated that proteomic patterns based on such profiles can distinguish active TB from healthy and symptomatic controls [12].

In the present study we hypothesized that plasma proteomic differences would also distinguish patients with active TB from those without active TB but with overlapping clinical symptoms, irrespective of the co-existence of LTBI. Here we show that using this approach, we can indeed discriminate accurately between such patient groups.

## Methods

### Ethics Statement

All participants gave written informed consent and the research was approved by internationally accredited ethics committees including Universidad Peruana Cayetano Heredia (Lima, Peru) and Imperial College London (London, United Kingdom). The study involved adults from 15 years of age. Informed consent was obtained from the next of kin, carers or guardians on the behalf of the young adults involved in the study.

### Study Participants

**Figure 1:**
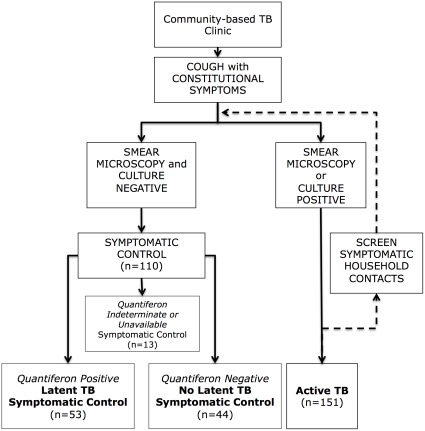
Patient recruitment. The figure illustrates the definitions of the patient subgroups and the routes by which they were recruited into the study.

Participants were recruited over a period of two years from adults over the age of 15 years attending 16 community TB clinics serving a population of ∼400,000 in the shantytown of Ventanilla on the outskirts of Lima, Peru (Figure 1). All patients underwent the local standardized clinical workup for TB. This included up to 4 consecutive sputum samples for microscopy and culture. Participation in the study did not change patients’ routine clinical management. The local incidence of TB in this population is ∼130 per 100,000/year [16] and 95–97% of TB cases are HIV negative [17]. We recruited patients with active TB and individuals, termed symptomatic controls, presenting with respiratory symptoms suspicious of TB in whom TB was subsequently excluded.

### Definition of Active TB, Latent TB and Symptomatic Controls

Active TB cases were recruited on the basis of positive sputum microscopy with subsequent confirmation by culture. Mycobacterial culture was by automated liquid culture (BACTEC MGIT 960™, BD) as well as the Microscopic Observation Drug Susceptibility (MODS) assay which we have previously established as a standard local laboratory protocol [18] and which has since been adopted as the standard operating procedure by the national TB programme in Peru. Symptomatic controls, those patients with respiratory symptoms without active TB, were recruited if they had a persistent cough and one or more of the following clinical features: fever, weight loss, decreased appetite or haemoptysis. Symptomatic controls had 1–4 sputum smears and cultures to exclude active TB and were followed for 6 months to confirm that cultures had not become positive or were re-classified accordingly. Additional TB cases and symptomatic controls were identified through tracing household contacts, from whom sputum smears and cultures were obtained if symptomatic.

**Table 1:** Characteristics of study patients.

	Active TB	Symptomatic Controls		
		Latent	No Latent	All
**N (%)**	151	53 (48)	44 (40)	110**
**Age Years**Median (IQR)	28.5 (15.5)	37 (27.5)	29 (20.5)	32 (23)
**Sex Ratio**Female:Male	68∶83	34∶19	30∶14	73∶37
Smear***				
Positive	139	0	0	0
Negative	7	53	44	110**
**Culture^∧^**				
Positive	139	0	0	0
Negative	8	53	44	110**
**History BCG vaccination** (%)	121 (80)	44 (83)	39 (89)	94 (86)
**Previous history** **TB** (%)	34 (22)	11 (21)	6 (14)	20 (18)
**Tuberculin Skin** **Test** (%)^†^				
Positive	*	33 (62)	13 (30)	46 (42)
Negative	*	11 (21)	25 (57)	36 (33)
**Cough>7 Days** (%)	118 (78)	24 (45)	22 (50)	53 (48)
**Haemoptysis** (%)	72 (48)	15 (28)	11 (25)	28 (26)
**Fever>7 Days** (%)	40 (26)	4 (8)	1 (2)	6 (6)
**BMI** Mean (sd)	21.6 (3)	25 (4.8)	23.5 (4.3)	24.1 (4.7)
**Night-sweats>** **10 Days** (%)	53 (35)	7 (13)	4 (9)	14 (13)
**Weight-loss>** **4 Weeks** (%)	69 (46)	9 (17)	5 (11)	19 (17)

TST-Tuberculin Skin Test. BMI-Body Mass Index. *TST was not performed in patients with active TB. **The QuantiferonGold test was indeterminate or unavailable on 13 symptomatic control patients. ***Smear results from 5 patients were unavailable. **^∧^**Culture results from 4 patients were unavailable. ^†^TST results were unavailable for 28 control patients.

An IFN-γ Release Assay (IGRA) (QuantiFERON-TB Gold In-Tube®) was performed on all participants. Latent TB was defined as a positive QuantiFERON® assay in the absence of clinical or microbiological evidence for active TB.

The Tuberculin Skin Test (TST) has limited value in the diagnosis of active TB and it was not carried out in our active TB patient group. We carried TST in the symptomatic controls group.

### Sample Collection

A 4 ml blood sample was obtained from each participant in an EDTA blood collection tube for subsequent plasma separation. Three additional aliquots were obtained at the same time for the QuantiFERON®-TB Gold in tube assay. Plasma was obtained before initiating TB treatment; otherwise plasma was taken within 1–2 days of treatment. Blood samples were transferred to the central laboratory on ice. Plasma was separated (3500 rpm, 10 minutes), aliquoted and frozen at −70°C at 6 hours following collection.

**Figure 2:**
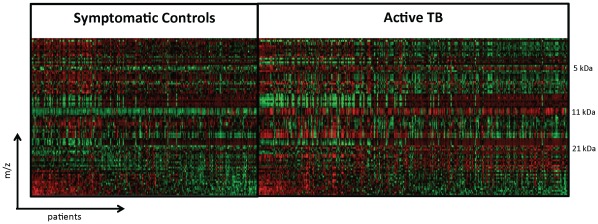
Heat map of crude plasma spectral data from active TB and symptomatic controls. Each vertical line represents an active TB patient or symptomatic control. Each horizontal line represents a protein with a particular molecular mass. Areas where a protein is present in high abundance are seen in red and low abundance in green.

**Figure 3:**
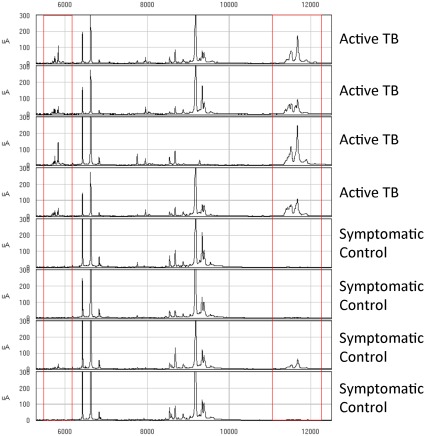
Mass spectra comparing 11.5 kDa and 5.8 kDa peaks in active TB and symptomatic controls. Mass spectra from 5 kDa to 12 kDa of four active TB and four symptomatic controls individuals. Intensity in µA is plotted in y-axis.

QuantiFERON® -TB Gold in Tube Assay

This was performed according the manufacturer’s instructions (Cellestis Plc, Sydney, Australia).

### Plasma Proteomic Profiling

Plasma was profiled using Surface Enhanced Laser Desorption/Ionisation-Time Of Flight (SELDI-TOF) mass spectrometry. All samples underwent a single freeze-thaw cycle prior to analysis. Samples were coded, blinded and randomised before application onto weak cation exchange (CM10) ProteinChip® arrays (Bio-Rad) in duplicate, as previously described [12]. Each ProteinChip® included 1 quality control standard derived from a single healthy individual, placed at random. Liquid handling steps were automated using a Biomek 3000 Laboratory Automation Workstation (Beckman Coulter) and a 96 well Bioprocessor® (Bio-Rad).

**Figure 4:**
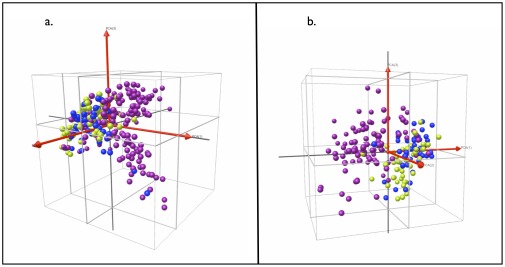
Clustering of patients with active TB and symptomatic controls with or without latent TB using principal component analysis. a. Crude plasma spectra; b. Fractionated plasma spectra. Each sphere represents an individual patient spectrum plotted in 3D space defined by the first three principal components. Purple = active TB; Blue = symptomatic controls with latent TB; Green = symptomatic controls without latent TB.

Mass spectra were generated on an automated System 4000 Bio-Rad ProteinChip® reader. Mass spectra data were collected and analysed using the ProteinChip® Data Manager Client 3.5 software (BioRad Inc.). Spectra were generated at both high (3,000 nJ) and low (1,600 nJ) laser energies with mass focus set to 40,000 Da and 6,000 Da respectively. Spectra were normalised by total ion current starting with a minimum mass/charge (*m/z*) of 2,500. Spectra with normalisation factor outside mean ±2 standard deviations were removed. The remaining spectra were re-normalised by total ion current. Spectral peaks corresponding to mass/charge (*m/z*) clusters were detected and clustered using the ProteinChip® Data Manager Client 3.5 software (BioRad Inc.) by auto-detecting peaks to clusters in two steps. For the first step a signal to noise ratio of 5 and valley depth of 3 were used, with a minimum peak threshold of 20% of all spectra. For the second step a signal to noise ratio of 3 and valley depth of 1 were chosen. The cluster window was set at 1.0 peak width and expression difference mapping performed over *m/z* range of 2,500 to 200,000.

Instrument calibration was performed using All-in-1 Peptide and Protein calibrants (Bio-Rad). Reproducibility was determined by measuring the inter-ProteinChip® coefficient of variation (CV) for the quality control spectra, based on all peaks in the spectrum with intensity >1 µA. Overall interchip CV for the quality control sample was 20%, consistent with similar studies.

### Plasma Anion Exchange Fractionation

Because highly abundant proteins/peptides suppress signal from lower abundance analytes in complex mixtures such as crude plasma, SELDI-ToF spectra were generated from both crude and pre-fractionated plasma to determine whether accessing the ‘deeper’ proteome yielded additional diagnostic information. Anion-exchange fractionation was carried out using the ProteinChip® Serum Fractionation Kit (Bio-Rad) according to the manufacturer’s instructions with a Biomek 3000 Laboratory Automation Workstation. Six fractions were obtained from each sample eluting at pH 9.0, pH 7.0, pH 5.0, pH 4.0, pH 3.0 and organic phase.

**Figure 5:**
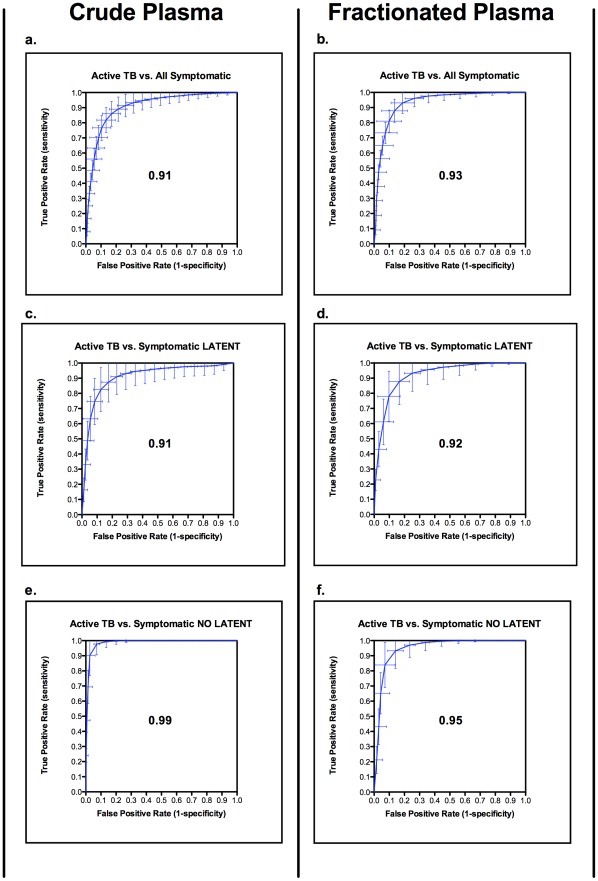
Diagnostic performance of proteomic fingerprints. The diagnostic performance of classifiers based on proteomic fingerprints are shown using Receiver Operator Characteristic Curves (ROC). (a,b) active TB *vs*. all symptomatic controls using crude or pre-fractionated plasma respectively; (c,d) active TB vs. symptomatic controls with latent TB using crude or pre-fractionated plasma respectively; (e,f) active TB vs. symptomatic controls without latent TB using crude or pre-fractionated plasma respectively. The ROCs are derived from 1000 random train/test re-samplings of the data. Error bars show standard deviations. The Area Under the Curve (AUC) is shown in the centre of each plot.

### Data Analysis

To visualize the covariance within the mass spectral profiles we used Principal Component Analysis (PCA). PCA encapsulates the covariance within a set of variables by extracting a ranked set of independent factors or principal components. The first 3 components encompass a high proportion (∼95%) of the informational content of a multivariate dataset. We plotted each patient with respect to the first 3 components, in 3-dimensional space, color-coding according to patient group.

Although PCA is useful for visualizing data it cannot provide a classification rule for discriminating between patient categories. To find such discriminatory proteomic patterns, we adopted a supervised learning approach in which patient categories are used to train an algorithm to derive a classification rule. We used a Support Vector Machine (SVM) method [19]. Briefly, we used 10-fold cross validation to select parameters for the SVM. For the final model parameters, we selected those that gave the overall highest accuracy across the whole 10 fold cross validation. We next selected a subset of the most relevant mass clusters using the Recursive Feature Elimination (RFE) algorithm [20] which ranks variables based on their contribution to the classifier. To obtain accuracy estimates for the classifier, we took 1000 random re-samplings of the original data, using 90% for training and 10% for testing. We selected as a final classifier the one that produced the highest accuracy while requiring the smallest number of *m/z* clusters. Results were expressed as sensitivity, specificity and accuracy (proportion of correct classifications) and as Receiver Operator Characteristic (ROC) curves. We assessed the different performances of classifiers derived from crude and pre-fractionated plasma by comparing mean values for sensitivity, specificity and accuracy using unpaired 2-tailed t tests. Comparisons of categorical data were by Fisher’s exact test.

**Table 2:** Discrimination of active from latent tuberculosis in symptomatic patients.

	Accuracy (%±sd)	Sensitivity (%±sd)	Specificity (%±sd)	AUC±sd
**Crude Plasma: Clinical Group** (N)				
**ActiveTB** (151) **vs. All Symptomatic Controls** (110)	85±7	85±9	84±10*	0.91±0.06
**ActiveTB** (151) **vs. Symptomatic LATENT Controls** (53)	88±7	92±7	75±19**	0.91±0.08
**ActiveTB** (151) **vs. Symptomatic NOLATENT Controls** (44)	95±5	96±5	91±14	0.99±0.02
				
**Pre-fractionated Plasma: Clinical Group** (N)				
**ActiveTB** (99) **vs. All Symptomatic Controls** (100)	87±7	84±12	90±10*	0.93±0.06
**ActiveTB** (99) **vs. Symptomatic LATENT Controls** (49)	87±9	89±10	82±18**	0.92±0.08
**ActiveTB** (90) **vs. Symptomatic NOLATENT Controls** (40)	90±8	90±10	92±13	0.95±0.06

The classifier performance is expressed as accuracy, sensitivity and specificity as percentages +/−standard deviations obtained by 1000 train/test randomizations of the data. (AUC) = Area Under Curve in ROC analysis. *Pre-fractionated *vs.* Crude Plasma p<0.001. **Pre-fractionated *vs.* Crude Plasma p<0.001. For all other comparison there are not significant differences between the performance of crude and pre-fractionated plasma.

**Table 3:** Number of mass/charge (m/z) clusters derived from crude and pre-fractionated plasma.

	Crude Plasma	Pre-fractionated Plasma					
		F1	F2	F3	F4	F5	F6
**ActiveTB vs. All Symptomatic Controls**	271 (98)	102 (10)	72 (8)	93 (4)	85 (8)	75 (12)	96 (12)
**ActiveTB vs. Symptomatic LATENT Controls**	271 (33)	102 (0)	72 (0)	93 (0)	85 (0)	75 (4)	96 (0)
**ActiveTB vs. Symptomatic NOLATENT Controls**	271 (57)	102 (16)	72 (0)	93 (8)	85 (0)	75 (8)	96 (5)

Total number of mass/charge (m/z) clusters obtained from SELDI-ToF profiling of crude and pre-fractionated plasma. In brackets number of relevant discriminatory m/z clusters selected by the RFE algorithm. F1 = fraction 1 at pH 9; F2 = fraction 2 at pH 7; F3 = fraction 3 at pH 5; F4 = fraction 4 at pH 4; F5 = fraction 5 at pH 3; F6 = fraction 6 organic phase.

## Results

### Characteristics of study patients

151 patients with active TB and 110 symptomatic controls were recruited (Figure 1). Of patients with active TB, 139 were both smear and culture positive, with the remainder either smear or culture positive. 48% of symptomatic controls had LTBI on the basis of a positive QuantiferonGold assay. Symptomatic controls had clinical features overlapping those of active TB patients, including cough, haemoptysis, fever, night sweats and weight loss, although symptom duration was generally longer among TB patients. Similar proportions of TB patients and symptomatic controls reported a previous history of TB (22% *vs.* 18%). The proportion reporting a history of TB was higher among controls with LTBI than among those without but did not reach statistical significance. Patients with active TB had lower BMIs at the time of recruitment compared with symptomatic controls (21.6 *vs.* 24.1 p<0.001). As expected, a higher proportion of patients with LTBI based on a positive IGRA had positive TSTs (>10 mm) compared with those without LTBI (62% *vs.* 30%, p<0.001). There was a higher proportion of female patients among the symptomatic controls than among the TB group. The effects of this potential bias are discussed below. Other key clinical features of the participant groups are given in Table 1.

### Discrimination of Active from Latent Tuberculosis in Symptomatic Patients

We plotted crude plasma global protein expression profiles in a heat map (Figure 2) that shows spectra patterns from active TB patients and unhealthy controls. The most striking area of up-regulation in TB patients is seen in the 11 kDa region where a series of protein peaks are seen in red amongst TB patients (Figure 2). A parallel area of up-regulation is seen at 5 kDa and a third smaller area seen at the 21 kDa region (Figure 2). Inspecting in more detail the spectra in the 5.8 and 11.5 kDa regions (Figure 3) reveals a complex of peaks at both these regions, which is more abundant in patients with active TB.

We assessed overall separability of patient groups by PCA of mass spectra from crude and pre-fractionated plasma (Figure 4 a–b). In figure 4, each patient sample is plotted in a 3-dimensional space defined by the first 3 principal components. The spectra from patients with active TB (purple spheres) cluster relatively tightly together and are well separated from symptomatic control patients (blue and green spheres) regardless of LTBI. This analysis, however, does not clearly separate symptomatic controls with or without LTBI (blue and green spheres, respectively).

The SVM classifiers distinguished active TB from both classes of symptomatic controls. The ROC curves in Figure 5 (a–f) summarize the performance of the classifiers, in terms of the trade-off between sensitivity and specificity, for each of the different comparisons. In each case, the area under the curve (AUC) exceeded 0.9, irrespective of whether crude or pre-fractionated plasma was analyzed, indicating a high level of discrimination. Tables 2 and 3 and Tables S1 and S2 summarize the performance of the classifiers in discriminating active from latent tuberculosis in symptomatic patients using the number of selected relevant m/z clusters (Table 3 in brackets). It was possible to distinguish patients with active TB from undifferentiated symptomatic controls with partially overlapping respiratory and constitutional symptoms with an overall accuracy of 85% using crude spectra with 98 relevant *m/z* clusters (Table 2, Table 3, Figure 5a). A higher specificity for active TB (90% vs. 84%, p<0.001) was achieved using pre-fractionated plasma with a total of 54 relevant *m/z* clusters (Table 2, Table 3, Figure 5b). Notably, these levels of discrimination were achieved despite nearly half of the symptomatic controls having LTBI (Table 1).

To further investigate the influence of background LTBI on classifier performance, separate comparisons were made between active TB and symptomatic controls either with or without LTBI. In both comparisons, active TB could be distinguished from symptomatic controls with overall classifier accuracies of at least 87% (Table 2, Table 3, Figure 5 c–f, Tables S1 and 2). Active TB was readily distinguishable from symptomatic controls without LTBI using both crude and fractionated plasma, with overall accuracies, sensitivities and specificities of at least 90% (Table 2, Table 3, Figure 5 e,f and Table S1 and S2). The main influence of LTBI among the symptomatic controls was to reduce classifier specificity, reflected in a higher proportion of false positives. Strikingly, plasma pre-fractionation improved specificity from 75% to 82% only using four m/z clusters (Table 2, Table 3, Figure 5 c,d, p<0.001).

To address the issue of the gender bias in cases and controls we reanalysed the data to determine whether a classifier based on the proteomic profile could reliably discriminate males from females. This was found not to be the case, suggesting that gender is not a major confounder in our analysis. As a further test, a new classifier was trained on male patients alone, to discriminate active TB from symptomatic controls. When we applied the trained classifier to the female subjects, this classifier was nevertheless still capable of classifying TB to an accuracy of approximately 80%.

We also confirmed the presence of differential expression of the Serum Amyloid A (SAA, 11.5–11.8 kDa) and transthyretin (13.7–13.8 kDa ) peak complexes which emerged in our previous study [12] as important informative markers for active TB. SAA was identified by specific immunodepletion (data not shown).

## Discussion

In this study we have shown that a distinctive pattern of plasma proteins distinguishes patients with active TB from non-TB patients with overlapping clinical features, even in the presence of LTBI. This both reinforces and substantially extends our previous findings where we first showed that proteomic patterns could be used as a diagnostic approach for active TB [12]. We have now shown that the proteomic pattern does not merely reflect the presence of TB infection *per se.* Rather, it can be used to identify active TB even in a highly TB-endemic setting with high prevalence of both respiratory symptoms and background LTBI.

The ability to discriminate rapidly in a symptomatic patient between active TB and non-tuberculous disease has profound implications for both individual clinical management and TB control programs [21]. For example, current diagnostic limitations frequently result in many patients in resource-poor settings being treated empirically for community acquired pneumonia before eventual diagnosis of active TB. This may lead to on-going transmission during the interval preceding diagnosis as well as greater individual morbidity. The alternative strategy of empirical anti-TB chemotherapy is sometimes employed, but cost, toxicity and logistics often preclude this. Adjuncts to conventional microbiology for diagnosis of active TB in widespread use include the TST and IGRAs. The use of TSTs in the diagnosis of active TB in high prevalence settings is greatly limited by its poor specificity for active TB as reactivity is also seen in LTBI, previous BCG vaccination and exposure to environmental mycobacteria. Nor has the recent introduction of IGRAs into clinical practice resolved this key diagnostic issue. This is because of their inability to distinguish active TB from LTBI [6] and frequent false negative results in acute active TB [22], limitations which are especially problematic in high prevalence settings [23]. Thus a diagnostic that overcomes these limitations is urgently required and would be a major advance in the management of the global TB pandemic. Recently it has been reported that a TNF-alpha^+^ TB-specific CD4+response can be used to differentiate latent infection from active TB but the sensitivity was just 67% [24]. Moreover, that study relied on polychromatic flow cytometry limiting the feasibility of being translated in high prevalence settings. In contrast, our approach provides improved accuracy, 87%, by detecting relevant protein biomarkers in plasma. Despite the discovery-phase of our approach using sophisticated proteomic methodologies, the identification of relevant plasma proteins leads to a clear translational path for antibody-based point-of-care devices that can be used to measure these plasma proteins in the future.

There is increasing interest in the identification of novel biomarkers for TB - in the contexts of diagnosis, treatment response monitoring, prediction of relapse or re-activation and as surrogates for vaccine protection. Most studies have focused on individual markers such as secreted *M. tuberculosis* antigens, serological responses, microbiological indices and host inflammatory markers, with mixed results [7], [25]. There is growing recognition of the advantages of using combinatorial biomarker panels or ‘omics’-based methods to achieve sufficient levels of accuracy [25]. However, relatively few studies have utilized such strategies.

Proteomic fingerprinting for biomarker discovery has been applied in the past decade to a variety of disease states, particularly in the sphere of cancer diagnostics [26], [27]. The power of this approach is reflected by the recent granting of FDA approval of a novel blood test derived from a SELDI-based fingerprinting method, for distinguishing malignant from benign ovarian tumours [27], [28]. In many infectious diseases, there are clinically important distinctions to be made between different manifestations associated with the same underlying pathogen. For example, distinguishing colonization or latent disease from active infection has obvious clinical and therapeutic implications. TB is a clear case in point. Proteomic fingerprinting has enormous potential for defining and distinguishing these disease states but has only recently received attention in this area [12]–[14], [29]–[31]. Because the circulation samples deep tissues throughout the body, local proteomic changes in organs such as the lungs can be reflected in the plasma proteome. Moreover, host modulation by the pathogen is likely to generate changing patterns of protein expression associated with different clinical manifestations. Thus the plasma proteomic response is a plausible index of disease state. Proteomic patterns are highly dynamic and it may be possible to define those that reflect stages in progression from latency to active disease. However, the complexity of the plasma proteome with its enormous dynamic range of solute concentrations means that detection of informative lower abundance proteins is particularly challenging. It is possible that differences between active TB and LTBI in symptomatic patients are reflected better by such lower abundance proteins not easily detectable in crude plasma. This may explain the higher specificity for active TB obtained from pre-fractionated as compared to the crude plasma spectra.

The gold standards used for defining patient groups in this study are notoriously imperfect. For example, while active TB was defined by positive microbiology, it is possible that some patients designated symptomatic controls may actually have had smear and culture negative TB. This might have resulted in an underestimate of the specificity of our diagnostic pattern for active TB, although our 6 months follow-up and appropriate re-labelling should have identified most of these. The lack of an adequate gold standard for defining LTBI must also be considered. While IGRAs show greater specificity than TSTs, sensitivity may be compromised especially in early active TB [22]. Thus some patients with unrecognized smear and culture negative TB may have been mislabeled as symptomatic controls without LTBI.

We did not perform routine HIV testing in our patient cohort and it is possible that over-representation of HIV seropositivity in our active TB group may have had a confounding effect. We believe this is unlikely in view of the low prevalence of HIV co-infection among TB patients in Peru (<5%) found in previous studies [17]. Important areas of future study will be to establish the applicability of this approach in the contexts of TB-HIV co-infection and smear-negative TB.

Our present findings confirm the utility of defining the host proteomic response in distinguishing clinically overlapping patient groups in a TB clinic setting. Moreover, this study shows that active TB can be identified by a blood test in a population of community TB clinic attenders, on a background of non-TB attributable symptoms, despite the coexistence of LTBI. Ultimately, a significant impact on control of TB in high prevalence settings will depend on the ability to translate these findings into a robust, affordable point-of-care format. Incorporation of a panel of biomarkers derived from this study into a lateral flow device or similar platform is the logical next step. Finally, the utility of defining proteomic patterns in TB may extend beyond diagnostics to provide new methods for monitoring treatment response and disease stage.

## Supporting Information

Table S1 **Selected relevant m/z clusters from crude plasma.**
(XLS)

Table S2 **Selected relevant m/z clusters from pre-fractionated plasma.**
(XLS)
